# “Now I Am Myself”: Exploring How People With Poststroke Aphasia Experienced Solution-Focused Brief Therapy Within the SOFIA Trial

**DOI:** 10.1177/10497323211020290

**Published:** 2021-06-15

**Authors:** Sarah Northcott, Alan Simpson, Shirley Thomas, Rachel Barnard, Kidge Burns, Shashivadan P. Hirani, Katerina Hilari

**Affiliations:** 1 City, University of London, London, United Kingdom; 2 King’s College London, London, United Kingdom; 3 University of Nottingham, Nottingham, United Kingdom; 4 Expert Independent Practitioner, London, United Kingdom

**Keywords:** brain injury, communication, community and public health, hope, psychology, psychological issues, aphasia, stroke, therapies, well-being, qualitative, framework analysis, England

## Abstract

Aphasia, a language disability, can profoundly affect a person’s mood and identity. The experiences of participants who received Solution-Focused Brief Therapy, a psychological intervention, were explored in the Solution-Focused brief therapy In poststroke Aphasia (SOFIA) Trial. Thirty participants with chronic aphasia, 14 with severe aphasia, participated in in-depth interviews that were analyzed using framework analysis. Two overarching themes emerged: valued therapy components (exploring hopes, noticing achievements, companionship, sharing feelings, and relationship with therapist) and perceptions of progress (mood, identity, communication, relationships, and independence). Participants were categorized into four groups: (a) “changed,” where therapy had a meaningful impact on a person’s life; (b) “connected,” where therapy was valued primarily for companionship; (c) “complemental,” where therapy complemented a participant’s upward trajectory; and (d) “discordant,” where therapy misaligned with participants’ preference for impairment-based language work. This study suggests that it is feasible to adapt a psychological therapy for people with aphasia, who perceive it as valuable.

## Introduction

Around 30% of people who have a stroke experience aphasia (Flowers et al., 2016), a language disability that affects speaking, understanding, reading, and writing. Acquiring aphasia can profoundly disrupt a person’s identity, life plans, and hopes for the future (Bright et al., 2013; Shadden, 2005). Aphasia also challenges a person’s ability to maintain a diverse social network (Northcott, Marshall, & Hilari, 2016) and can lead to people having fewer friends and engaging in fewer social activities (Cruice et al., 2006; Northcott, Moss, et al., 2016). Depression and low mood are common sequelae of aphasia (Baker et al., 2020; Hilari et al., 2010; Kauhanen et al., 2000). It is therefore a concern that there is currently limited evidence for effective interventions to address the psychological well-being of people with aphasia (Baker et al., 2018). Furthermore, it is not well explored how people with an acquired communication disability experience psychological interventions. This article investigates the experiences of people with poststroke aphasia who received an adapted form of Solution-Focused Brief Therapy (SFBT).

SFBT is a psychological therapy that explores a person’s resources and expertise rather than focusing on their deficits (de Shazer et al., 2007; Ratner et al., 2012). The strongest evidence for its effectiveness is with adults with depression (Gingerich & Peterson, 2013). A recent meta-analysis of the use of SFBT in medical settings reported a significant effect of SFBT (*d* = 0.34, *p* < .05) for health-related psychosocial outcomes (Zhang et al., 2018). Within stroke research, a trial reporting on 62 working-age people (≤65 years) with mild-moderate first stroke found significant benefit in terms of better mood and lower anxiety for the intervention group who received 10 SFBT sessions shortly after hospital discharge, compared with the control group, who received usual care (Wichowicz et al., 2017).

There is concern that due to their language disability, people with aphasia are often excluded from receiving mental health interventions (Baker et al., 2021; Northcott et al., 2017) and from taking part in psychological stroke research (Townend et al., 2007). For example, the only stroke trial of SFBT excluded people with aphasia on the grounds that they would not benefit due to the linguistic and cognitive demands of the therapy approach (Wichowicz et al., 2017). Whereas it has been reported that people with aphasia appear to receive little psychological therapy and would like to be offered more (Baker et al., 2020), it is less well explored how they experience receiving a psychological therapy when it has been adapted to be accessible for them. This study explores the perspectives of people with aphasia who were offered SFBT within the context of the SOlution Focused brief therapy In poststroke Aphasia (SOFIA) Trial, a feasibility randomized controlled trial with wait-list design (Northcott et al., 2019). The trial builds on a previous proof-of-concept study with five people with mild-moderate aphasia, who reported finding SFBT highly acceptable (Northcott et al., 2015).

It is increasingly recognized that including a qualitative component within a randomized controlled trial is desirable, particularly when evaluating complex health interventions (Craig et al., 2008; Lewin et al., 2009). Qualitative research can provide insight into contextual and individual factors that may influence how someone responds to a complex intervention, and explore the processes underlying a reported effect (Johnson & Schoonenboom, 2016). Furthermore, a better understanding of the variation in individual responses and outcomes in a feasibility trial may improve the design and implementation in a future definitive trial (O’Cathain et al., 2013).

The aims of this study were to explore the following:

**Research Question 1 (RQ1):** How do people with aphasia experience receiving SFBT?
**Research Question 2 (RQ2):** What is the perceived value of the intervention to participants?
**Research Question 3 (RQ3):** What factors lead people to respond differently to the intervention?

## Method

This qualitative research was embedded within the SOFIA Trial (ClinicalTrials.gov Identifier NCT03245060). SOFIA was a single-blind, randomized, wait-list-controlled feasibility trial. It aimed to explore the acceptability of SFBT for people with aphasia and assess the feasibility of conducting a future definitive trial determining the effectiveness of the approach to enhance well-being (Northcott et al., 2019). Quantitative results for the SOFIA Trial are reported elsewhere. Reporting of qualitative findings adheres to the Standards for Reporting Qualitative Research guidelines. Ethical approval was granted by the National Health Service (NHS) Health Research Authority, Brighton and Sussex Research Ethics Committee (17/LO/1255). Local NHS Research and Development approvals were gained from participating sites. All participants gave written informed consent. Pseudonyms, replacement terms, and vaguer descriptors are used throughout this article to preserve anonymity.

Participants were either randomized to the intervention group (started intervention immediately post-randomization) or to the wait-list group (started intervention 6 months post-randomization). Although SFBT is often brief (three to five sessions; Ratner et al., 2012), it was anticipated that people with aphasia might benefit from additional sessions as their language disability can mean it takes longer to cover material (Northcott et al., 2015). Therefore, participants were offered up to six sessions. Typically, ownership of ending SFBT therapy rests with the client (Ratner et al., 2012). SOFIA participants could elect how they spaced sessions within a 3-month window and were invited to have as many of the six sessions as they perceived would be useful. Therapy visits took place either in participants’ homes or the university clinic. Interviews with the intervention group took place 6 months post-randomization, so approximately 3 months after the intervention finished. Interviews with the wait-list group took place 9 months post-randomization, soon after the final therapy session.

### Therapy Approach and Theoretical Model

Two main elements of SFBT are building up a picture of a person’s preferred future and inviting them to notice what is already working. The client is considered expert in their own lives, thus it is for the client to know their preferred outcome from the therapy, and for the therapist to enable them to find their own way forward, drawing on the person’s strengths, skills, and resources (de Shazer et al., 2007; Ratner et al., 2012). Within the SOFIA Trial, emphasis was also placed on acknowledging the difficulties and distress of living with stroke and aphasia. Family members were invited into therapy sessions if this was the preference of the person with aphasia. All three trial therapists were experienced speech and language therapists (SLTs). They received 6 days of initial training, as well as regular supervision, real-time support as needed, and a therapy manual.

SFBT is a language-based intervention, which typically relies on complex linguistic structures, for example, questions exploring hypothetical future states. To adapt the approach for people with aphasia, the therapists used total communication strategies, drawing on the person with aphasia’s communicative strengths, for example, writing keywords, using gesture, drawing, pictures, and objects in the environment (Pound et al., 2000). Particular attention was given to simplifying questions and supporting abstract concepts visually. Therapists were encouraged to deliver SFBT flexibly to enable people with aphasia to participate. There was an expectation that, for people with severe aphasia, therapists would focus on more accessible components of SFBT, such as using scales supplemented by pictures and celebrating recent successes through sharing photos. The SOFIA TIDieR checklist provides further information about the intervention (https://doi.org/10.25383/city.8058539.v1). An illustrative case study of SFBT is provided in online Supplemental File 1.

**Figure 1.**
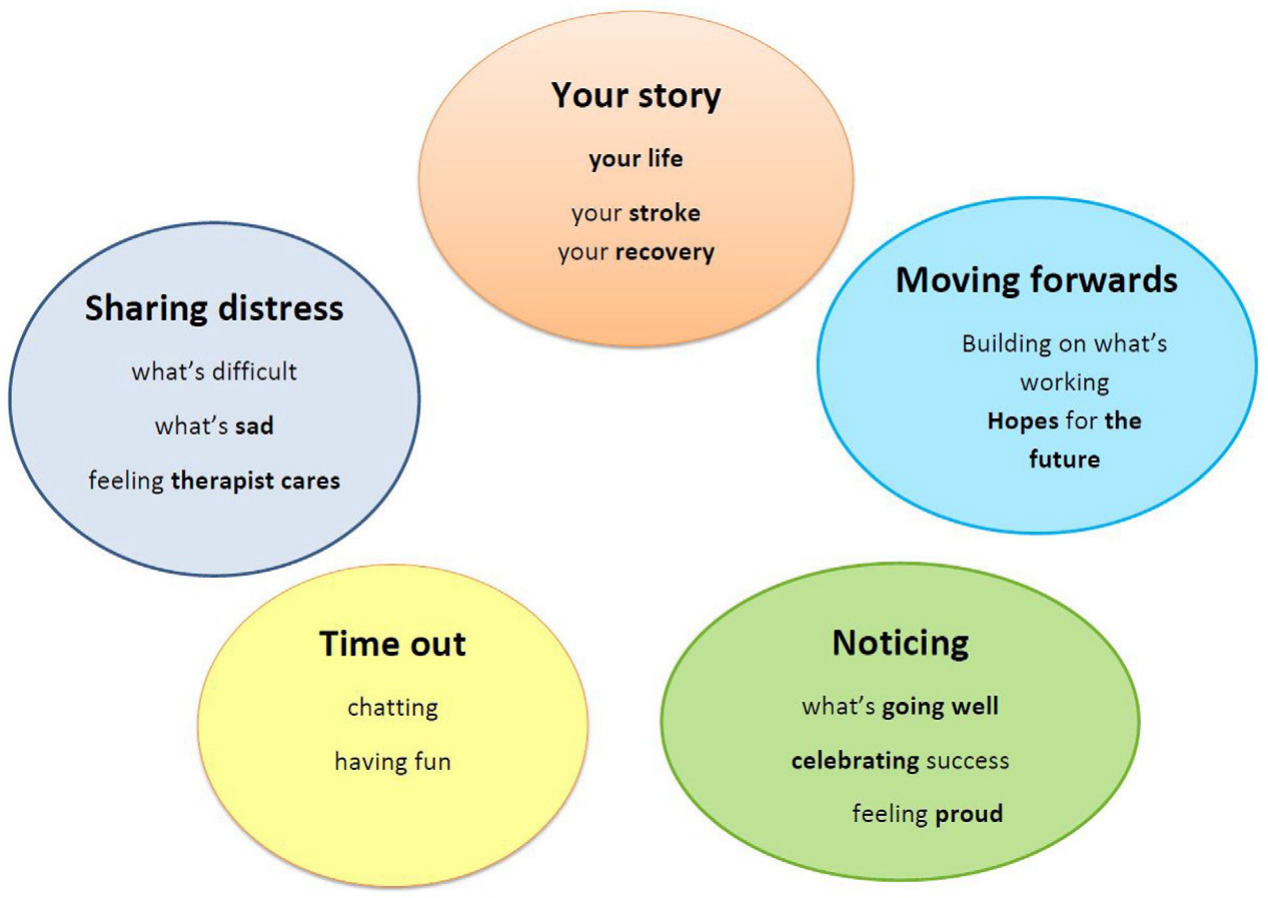
Schematic diagram of proposed therapy components.

The theoretical model underpinning how the SOFIA therapy was conceptualized to build change was the dual process model of bereavement (DPM; Stroebe & Schut, 1999). An individual’s response to stroke can be seen as a grief reaction, akin to other bereavement and losses, and recovery as a psychosocial transition, as a person adjusts to their new poststroke identity and life (Glass & Maddox, 1992). The DPM model describes how people come to terms with loss through loss-oriented and restoration-oriented work. An additional component is “time out,” where a person seeks respite from processing grief. Stroebe and Schut (1999) suggest that adaptive coping is brought about by oscillating between loss, restoration, and time out. The DPM model has been found helpful in capturing how people experience loss and adjustment following a stroke (Ch’Ng et al., 2008). We used the model to inform the core components of the intervention, as shown in Figure 1. Restoration work maps onto “Moving forward” and “Noticing,” grief work aligns with “Sharing distress” and “Your story,” and the final element is “Time out” (chatting, having fun).

### Participants

All participants who received the SOFIA intervention(*n* = 30) were invited to take part in the in-depth interviews. Through inviting all participants, we aimed to capture a diverse range of perspectives. To be eligible to participate in the SOFIA study, participants had a diagnosis of ischemic or hemorrhagic stroke, were at least 6 months poststroke, aged 18 years or older, and had aphasia as determined by the clinical judgment of an SLT. Participants with any severity of aphasia were included provided they had the mental capacity to consent to take part. Aphasia was assessed using the Frenchay Aphasia Screening Test (Enderby et al., 1987). Capacity was assessed both informally and through asking three simple yes/no or forced alternative questions, provided in an aphasia-accessible format, to confirm they had understood key aspects of the study (online Supplemental File 2). Exclusion criteria were as follows: having other diagnoses affecting cognition such as dementia or advanced Parkinson’s disease, severe uncorrected visual or hearing problems, severe or potentially terminal comorbidity, being in receipt of a psychological or psychiatric intervention at the time of recruitment, non-fluent English speaker prior to the stroke (based on self or family report), or not having mental capacity. Use of antidepressants or rehabilitation therapy was not a reason for exclusion, nor were participants excluded on the basis of their well-being or depression scores.

Participants were identified through two NHS Speech and Language Therapy services in London, United Kingdom. Potential participants were also identified through the community, for example, through visiting stroke groups organized by the voluntary sector.

### Procedures/Data Collection

The topic guide was developed by the first author (see online Supplemental File 3). It was based on an earlier version trialed in a pilot study with people with severe aphasia and refined through discussion with the project’s advisory group of people with aphasia. The topic guide did not include specific questions, but instead outlined topics to be explored in an organic way following participant responses. Topics included how participants experienced receiving the intervention; observations around logistics, dosage, and ending of therapy; and their reflections on any change that may have occurred. How they experienced study procedures was also explored and is reported elsewhere. To facilitate the person with aphasia, interviewers used the total communication strategies described above. They also referred to the schematic diagram of the therapy components (Figure 1) and picture resources, including some illustrating the content of their individual therapy.

All interviews were conducted in the participants’ choice of location. Most participants elected to be interviewed in their own home although four participants came into the university. All but two participants gave their consent for their interviews to be audio-recorded and these interviews were transcribed verbatim. For the two participants who declined to be recorded, the interviewer gained consent to make detailed notes that were typed up post interview. The content of these unrecorded interviews was analyzed alongside other interviews although quotes have only been used where the interviewer was able to record direct speech with accuracy. Of the 30 participants, seven were interviewed with a family member present for all or part of the interview, respecting the preference of the participant. The mean length of time taken to complete interviews was 48 minutes (range = 21–74 minutes).

There were three female interviewers, all of whom had extensive experience of working with people with aphasia and were experienced qualitative researchers. One of the interviewers was also a SOFIA therapist; to avoid biasing responses, she did not conduct interviews with participants where she had been the therapist. One of the interviewers had met all participants prior to the interview when consenting them into the trial; for all other interviews, the participant had not previously met the interviewer.

### Data Analysis

Data were analyzed using the “Framework” method (Ritchie & Spencer, 1994). Framework is increasingly used within qualitative health research as a flexible and systematic approach that can facilitate explanatory accounts and provide a clear audit trail from raw data through to final themes (Gale et al., 2013). Initially, the lead analyst read through all the transcripts to create a thematic index, adopting an inductive stance as opposed to using a predetermined framework. The index was further refined through discussion with the wider research team. A second analyst also read through six transcripts (20%), providing reassurance about the integrity of the index. All the material was then coded and a decision was made as to where it belonged within the thematic index. Thematic charts were constructed, with all material synthesized and placed in the appropriate cell of the relevant matrix. This matrix-based method of organizing the data enabled systematic exploration of the range and patterns of views and experiences and facilitated mapping of connections within and between cases. Although the initial coding was conducted by the lead analyst, a second analyst reviewed all the charted material and had access to all transcripts to cross reference the charts with the raw data. Both analysts then reflected on the emerging themes together. This resulted in refinements in how themes were conceptualized and provided reassurance that the material had been fairly represented and the diversity of experience captured.

The matrix-based system facilitated the development of a typology exploring the variation in how people experienced the therapy. The typology was multifactorial and thus a person was assigned to a category as determined by two key variables: (a) participant perceptions of the main value of the therapy, and (b) therapy-related change. Each participant was assigned to only one category. The second analyst independently categorized all participants within the proposed typology. For two participants, the second analyst was undecided. This was resolved through both analysts rereading the transcripts, further reflection and discussion, leading to a consensus decision. Data were managed using NVivo Version 12.

## Results

### Participants

Thirty-two participants were recruited into the SOFIA Trial. Of these 32 participants, 30 received the intervention and agreed to take part in an in-depth interview post intervention. Twenty-nine participants elected to receive all six sessions; one participant elected to receive five sessions. Sixteen participants were women and 14 were men; 43% were more than two years post stroke. The majority were White (73.3%), with 26.7% from Black, Asian, and minority ethnic backgrounds. Most lived with family members (60%). Sixteen participants had mild to moderate aphasia; 14 had severe aphasia (participants scoring <7/15 on either the receptive or expressive domains of the Frenchay Aphasia Screening Test categorised as ‘severe’). The following is presented online as supplementary material: participant characteristics (Supplemental File 4); individual participant profiles, grouped according to the developed typology (Supplemental File 5); a CONSORT diagram of the SOFIA Trial (Supplemental File 6); and prevalence of the different therapy components within participants’ accounts (Supplemental File 7).

### Main Findings

Two overarching themes emerged: (a) valued therapy components, and (b) perceptions around progress. There was variation in how people responded to the therapy. A typology is presented as a way of capturing patterns and to aid interpretation of the variation. Finally, participant reflections around receiving therapy in the context of a research study are described. These themes are displayed in online Supplemental File 8 and illustrated in the online Supplemental File 1 case example.

### Valued Therapy Components

Participants perceived the therapy sessions as conversations, rather than being therapist-led activities or exercises. There was a strong endorsement that the approach was suitable for people with aphasia, including severe aphasia. (“This experience, this, would to this, this, other, other people [with aphasia],” participant with aphasia). In terms of what participants valued about the conversations, five subthemes emerged: noticing achievements, encouragement to explore future hopes, explaining feelings and experiences, companionship and “time out,” and their relationship with the therapist. These are explored below. Box 1 illustrates how these different components complement each other.

**Box 1.** How the Therapy Components Work Together: A Case Example.

This case study illustrates how these five therapy components could complement one another as active ingredients in the therapy process. The participant, who had mild aphasia, described the companionable aspect of the therapy: “We got on well and oh we had a nice time . . . we got on like a house on fire [laughing].” This strong relationship underpinned conversations around hope, progress, and grief. She described how she found it “helpful, helpful,” to explore her hopes, for example, that one day she would again walk in her own garden. Interleaved into these future descriptions, she described her progress and achievements: “I told her about the things that I’ve learned, things that I’ve understood and so on . . . Because we talked a lot about how I am progressing, how I manage my walking and everything.” Throughout, the participant explained how the therapist listened to her distress. She shared with the therapist that she felt “Overwhelmed . . . things that I couldn’t seem to come to terms with. Oh, we did a lot of that.” She described how the therapist “Was here listening and taking part and joining in to all my conversations . . . oh yes, she was very good, helping me through the cold times . . . God helped me, and he helped [the therapist] in what she did.”

#### Being facilitated to notice personal qualities and achievements

Participants described how the therapy celebrated their successes. Sessions were a chance for them to share things that had gone well or that were good in their lives, including progress they were making poststroke. For example, one participant described how the therapist would make a list of “everything, everything, all lovely,” that she and her husband felt proud of, such as her gardening, cooking, and going to the gym. She described this process as “lovely . . . [therapist] was very good,” and agreed that it had helped her to notice positive things in her life.

When the therapist noticed participants’ achievements and personal qualities, this had a positive impact on their mood and could bolster their self-esteem and self-belief: “I have a lot of self-respect now . . . it changed um, my, my self-perception, my, my perception of myself got better.”

#### Encouragement to explore hopes for the future

It was perceived as helpful to talk through what a person wanted to be happening in the future. Within the therapy, participants described both their long-term ambitions (e.g, driving an adapted car) and goals which they achieved within the therapy block (e.g., buying gifts at a department store). Some participants made a direct link between these conversations and their motivation to do more:

Because I said I’m going to go to, erm, Dad’s house in [market town] and I said, and I, I want, erm, the train and I’ll do it, and I did . . . It was really good, I was proud.

Participants also had conversations around their hopes for future feeling states, such as feeling happier. They described how in therapy they talked through “just small things” that might help them to get there.

Many expressed a belief that it was important to look forward rather than backwards. It was not universal, however, that therapy included a future-focused component. An example is a participant who was frail with deteriorating health. She chose not to discuss the future, and instead she found it more valuable when therapy sessions focused on noticing what was going well and joking and having fun.

#### Feeling supported to explain how they feel

A common theme was that participants valued being enabled to explain how they were feeling. Within the therapy, feelings of sadness, panic, anger, or despair were shared. The context for some was that it was hard for them to have this conversation with others, often exacerbated by the aphasia. The feeling that someone understood their difficulties and experiences was perceived to make a difference as they felt supported instead of on their own. For example, one participant explained that in the therapy, “You can speak, you, you, to explain.” This contrasted with how he normally experienced conversations: “There’s the err, the panic, you, you, you, feel, the words with your mind, you, you racing, racing, yes, and is angry, angry . . . you can’t, you can’t speak, you, you can’t explain.” Post therapy, he described feeling less panicky, more confident, and better inside himself. He attributed this change to “explain, explained, yes, yes.” The interviewer clarified with him that he was referring to explaining about his feelings.

Not all participants reported that sharing distress and other difficult emotions was a part of the therapy, however. For participants who were not experiencing low mood or distress, this therapy component was perceived as less necessary. A further subset reported that, while they did sometimes feel sad or had difficult life situations, they had not wanted this to be the focus of therapy.They preferred to use therapy sessions as a distraction, or had a belief that it was not for others to solve their problems, or wanted to focus on future plans.

#### Companionship and “time out.”

The visits were a source of valued companionship, particularly for more socially isolated participants. A female participant in her 80s explained, “I haven’t got many friends because practically they are ill or they’re dead because everybody’s so ill, old you see, naturally.” She observed, “It’s nice, somebody, to see somebody . . . everything is something to see peoples.” Participants described how they valued having someone different to talk to when horizons had become narrower poststroke and to be able to talk about topics that were not discussed in everyday family or patient-carer interactions, such as stories from their family history.

For people with severe aphasia, the opportunity to express themselves and feel included in a conversation that was centered around them, and their life, was perceived as important. It was sometimes described as a relatively unusual occurrence:

Interviewer: And what was most important to you of all the things (in therapy)?

Participant: Mm, well, life.

Interviewer: Talking about your life?

Participant: Yeah, yeah.

Interviewer: Have you had to do that with many other people after your stroke?

Participant: Er, not really, not really.

Some participants reported that “time out,” including laughing and joking with their therapist, was the best part of the project. They described how they talked “about this and that and the other . . . we joked on the subjects,” and spoke about TV programs, children, laughed together at politicians, and sharing “tea, coffee.” For example, one female participant who had severe aphasia and limited mobility, explained how much she enjoyed chatting and sharing stories with her therapist (“She was lovely. I told her what I wanted, and we will look at things . . . oh I loved it, I loved it!”) and pointed to “Time Out” on the diagram (see Figure 1) to show what she had enjoyed most about the therapy.

#### Relationship with therapist

The relationship with the therapist appeared to be a key factor in how participants experienced the therapy. For example, a participant described his therapist as “delightful,” and the “best thing about [the study],” and agreed with his wife that he “enjoyed the therapy because he enjoyed [the therapist].” The relationship with the therapist was proffered as an explanation for how the therapy worked. This is illustrated by a participant who described the therapy as “uplifting.” When this was probed in the interview, she reflected, “[the therapist] is lovely person, isn’t she? So she, I think that’s what it was, you know, the way she spoke and she was gentle and she was nice.”

Participants reported getting on well with their therapist. They described being able to talk freely, feeling comfortable to share and disclose, and felt accepted by the therapist: “I felt comfortable with her, very nice . . . you could talk about anything to her.” It was common that interactions were perceived as two-way, with the therapist sharing from their own life. For example, one person described how the therapist was “Open and honest . . . it was a chance to speak to somebody who brought themselves.” The sense that their therapist cared about them, noticed their qualities, and gave them one-to-one time was perceived as important (“Something to make me somebody that cared . . . [made] a lot of difference.”). Several participants described feeling real affection for their therapist.

### Perceptions Around Progress

Participants rarely conceptualized their involvement in the project as leading to “change,” and not all participants were seeking to make changes. To describe the impact of the therapy, participants used words such as reinforced, mended, connected, improved, “a bettering,” and uplifted. These shifts were seen across the following domains: mood and identity, communication, relationships, independence, mobility, and participation. The next section reports on these domains as subthemes, before reporting on the final subtheme: perspectives around little or no change.

#### Mood and identity

The therapy was perceived by many to lift their mood. Participants described newly enjoying activities or feeling happier in themselves. An example is a participant who had her stroke 5 years previously. She described herself as depressed prior to therapy. The therapy helped her to “bring me out of myself.” She noted the difference this made to her mood: “I mean I’d wake up now sometimes and I smile, you know, because I’m glad to wake up, whereas . . . I didn’t want to wake up.”

The therapy was also seen as enhancing calmness, reducing stress and anxiety, and enabling people to feel more optimistic about the future (“At first I couldn’t feel good about the future but now I can look forwards not backwards.”). For a subset, the therapy was described as facilitating a renegotiation of poststroke identity. This could be in terms of increasing self-respect and noticing personal strengths. It could also enable people to connect with their sense of who they are.

It make you somebody, hey . . . oh, it good, good, good, good, and so, it give me courage, courage, courage . . . Now, now I am myself.

There was no one who attributed worsening of mood to participating in the project. However, there was variation between participants in how much their mood was affected by receiving therapy. There were participants whose mood improved during the study, but they attributed the change partially or wholly to other causes (e.g., support from family, improvements in speech, general poststroke recovery, and assessment visits within SOFIA). Conversely, external life events, such as deteriorating health, could impact on mood negatively. A further subset reported that they were cheerful both before and after therapy. Finally, there was a subset who reported low mood that was unchanged by the therapy.

#### Communication

A minority felt that the therapy had resulted in improvements in their talking, reading, or writing. More commonly, participants spoke about progress with talking coinciding with taking part in the project and attributed it to various factors, including the passage of time, having a positive outlook, or the combination of the different therapy inputs they had received. Nonetheless, a common theme was that participants described increased confidence to talk in different situations post therapy, such as speaking on the phone to family or having coffee with friends.

Persisting difficulties with talking were a cause of frustration and distress for many participants. This could color their view of their therapy gains. For example, one participant described his aphasia as “frustrating, I can’t tell you how frustrating it is.” The therapy had enabled him to speak more to friends, and resume playing bridge: “It’s given me more confidence. Yes, it’s, that’s helped me making, making me feel confident about talking with other people. And I haven’t found that before . . . [makes me] happier, happier.” Nonetheless, he expressed disappointment that the therapy had not improved his aphasia: “[The therapy] has given me a bit more confidence. But I suppose I am hoping [laughs] I suppose I’m hoping that someone’s going to give me, give me a miracle and it’s not going to happen.”

#### Relationships

Several participants spoke about seeing friends more. There were also changes to how some participants related to their grown-up children: They described feeling closer, increasing contact, and speaking more openly:

It helped me, for example, I start talk [on the phone] to my, my, my son [who lives abroad] . . . oh two years, two, two years I don’t, couldn’t do it, but [therapist] is there, I try to make it . . . my son is very happy now.

For a small subset of participants, the therapy caused positive shifts within the marital relationship. This is illustrated by a participant who was several years poststroke and had severe aphasia. She perceived that her husband had been protective of her following the stroke, for example, taking over the cooking. Following the therapy, her husband gave her more space and they became comfortable to give each other time apart. They renavigated their roles within the relationship, for example, she was doing the cooking again, even Sunday roasts, which she confirmed was “lovely, lovely.”

#### Independence, mobility, and participation

It was common that participants described increasing confidence with various activities of daily living post therapy, such as putting out the rubbish, getting dressed, food preparation, and managing to use a purse one-handed. Another theme was making progress with walking. Participants also described increased participation, such as going to restaurants, starting to volunteer, and using public transport. There was an acknowledgment of balancing risks, the need to be careful, and that progress could come in small steps. Feeling they were making progress was positive and could make someone feel proud: “If I’m good today to walk somewhere, yes, yes, for me it’s one victory, you know.”

Some directly attributed their progress to the therapy; for others, there were additional reasons, such as having as a new paid carer. It could also be hard to tease apart what was causing change (“Time or the sessions?”). There were persisting limits to participation due to poststroke physical disability, other comorbidities, and old age (“Not too much of going out, er, got a bit, er, worse . . . old age [laughing].”)

#### Not changing

A theme that emerged was that “change” was not what some participants wanted from the therapy. For this subset, the main value of the therapy appeared to lie in the warmth of the companionship and feeling noticed and valued as people by their therapist. This is illustrated by a participant who lived alone and characterized herself as being content with “the little things,” such as being able to go out independently and speak with friends. She had not wanted to make any changes in herself or her life prior to the therapy and reported that the therapy had not resulted in any change. Nonetheless, she felt strongly that the therapy was right for her and she valued it highly. She noted the therapy, “makes you happy because you, you talk to the person,” and felt a close bond with her therapist, “we’re human being . . . especially when you get to know each other, you get very close, don’t you?” She gave examples of the therapist noticing her skills, for example, in needlework, and appreciating what made her special as a person: “I was very pleased. She was pleased too. She said that you’re a remarkable woman.”

A subset, however, expressed disappointment that the therapy had not improved their aphasia. They would have preferred the focus of the therapy to be language exercises and discussed wanting more tangible therapy activities or worksheets. This is illustrated by a participant with severe aphasia who was 6 months poststroke when receiving the SOFIA intervention. His motivation for participating was to improve his talking. As such, he had hoped the focus would be, “Speech a little bit.” The therapy did not match what he was looking for: “I do like it, but it doesn’t me, it just doesn’t really . . . it wasn’t really what I wanted.”

### Who Benefits and Why? Development of a Typology

There was variation in how people experienced the therapy, what impact it had on their life, and which aspects of the therapy process they valued most. Participants were categorized into four groups (see online Supplemental File 5). The primary factor used to categorize participants was their perception of the main value of the therapy. Consideration was also given to their reflections on therapy-related change. Each group is defined below.

#### “Changed”: Meaningful impact (*n* = 11) “It give me courage . . . now, now I am myself.”

The therapy was highly valued and perceived to have made a meaningful difference in the participant’s life. This was either evidenced by increased participation in a variety of activities; improvements in mood, confidence, and self-perception; or a sense that the therapy had supported them through a difficult life situation. Pre-therapy, most participants in this group had low mood. Alternatively, the therapy matched an interest in making changes or reflecting on their lives. Participants’ initial motivation for participating in the project was not a defining factor: members of this group had a variety of reasons for participating, including finding a cure for their aphasia.

#### “Connected”: Connection and companionship (*n* = 10) “There is somebody come talk, and talking to you so you’re still alive, you know, still alive.”

This group was defined by valuing the therapy primarily for the companionship and the connection they felt with the therapist. Therapy facilitated little or no “change” although because few in this group wanted to make change this was not disappointing. Participants rarely had low mood prior to therapy although it was common that they felt isolated. Most would have liked more therapy sessions. Some found the ending of the therapy sad.

#### “Complemental”: Complementing an upward trajectory (*n* = 4) “I’m always going up . . . it’s going up, up, er, up, up, up.”

Participants were positive about the therapy. They all perceived that they were making progress and were on an upward trajectory. The SOFIA therapy was one contributory component. They typically described receiving other rehabilitation input immediately before or after the therapy received in SOFIA. It was hard for this group to disentangle whether their progress was due to SOFIA, other rehabilitation, or time.

#### “Discordant”: Dissatisfied with the focus of therapy (*n* = 5) “So like speaking, reading and writing, that’s really, really crux . . . um, how I feel, you know, I don’t care about that.”

Members of this group expressed disappointment with the therapy. Their aims for the therapy related to language and physical recovery, and they perceived that these aims were not met. Most did nonetheless like the therapy: They described a friendly, warm relationship with their therapist; found the sessions enjoyable or positive; and described some improvements typically in participation. Overall, this group perceived that the therapy did not fit well with what they wanted to focus on.

### Experiencing SFBT Within the Context of a Research Project

#### Motivations for participating in the study

Participants took part in the study mainly for four reasons: (a) *contribution*: participants described wanting to help others, wanting to give something back, wanting to enable researchers to better understand aphasia; (b) *companionship*: participants liked the idea of regular conversations; (c) *curiosity and interest*: participants were interested, curious about research, and liked feeling connected to the university; and (d) *progressing their talking*: participants hoped that taking part would mean receiving additional language therapy to improve their talking. Commonly, participants described a combination of these factors, with some suggesting that they took “any little thing” that was offered in case it turned out to be useful. Only one participant stated that she participated for support with her own emotional well-being.

#### Constraints on therapy offered due to study design

Some participants were satisfied with having six sessions (the upper limit) and considered this sufficient to enable a “bettering of my condition.” However, alternative perspectives included a preference for more sessions, having follow-up sessions, a more intensive schedule, or sessions spaced over a longer time frame. The rationale for wanting more sessions or follow-up sessions varied: Six sessions were perceived as insufficient and they wanted further sessions to support their progress; they enjoyed the sessions and so wanted them to continue indefinitely, particularly where they had developed a close relationship with the therapist; and they wanted to see the therapist from time to time to feel that someone cared.

## Discussion

The experiences of people with aphasia who received an adapted version of SFBT were explored in this study. Thirty participants took part in in-depth interviews and overwhelmingly reported that they found the intervention acceptable. Four main areas were identified as valued therapy components: exploring hopes for the future, noticing achievements, sharing feelings and experiences, and companionship. Underpinning all these components was the therapeutic relationship. Participants reported therapy-related change in areas such as improved mood and participation. The variation in how people responded to the therapy was captured through sectoring participants into four groups: (a) “changed,” where therapy was highly valued and had a meaningful impact on a person’s life; (b) “connected,” where therapy was valued primarily for the sense of connection and companionship; (c) “complemental,” where the therapy complemented a participant’s upward trajectory; and (d) “discordant,” where the therapy focus did not fit well with the participant’s preference for language-based work.

A striking finding from this study is that it was possible to adapt a psychological therapy to be accessible for people with a significant language disability: 47% of participants had severe aphasia. People with severe aphasia arguably have more need for psychological support than those with milder aphasia poststroke as they have worse quality of life (Hilari & Byng, 2009), and participate in fewer activities (Darrigrand et al., 2011). SFBT typically relies on cognitively and linguistically demanding tasks (Ratner et al., 2012). This study provides encouraging evidence that it is possible to adapt even a linguistically complex intervention to be accessible to people with aphasia. This counters the perception that people with aphasia, particularly severe aphasia, are unable to access psychological therapies due to the language demands (Wichowicz et al., 2017). Indeed, people with severe aphasia are represented in each category of the typology, suggesting that severity of aphasia was not a determining factor in how people responded to the intervention.

One factor that may have facilitated the inclusion of people with severe aphasia is that the therapy was delivered by SLTs. There has been recognition that psychological care is the responsibility of all health care professionals (Kneebone, 2016; Scott & Barton, 2010). A stepped care model suggests that it may be appropriate for specialist stroke professionals, such as SLTs, to deliver brief psychological interventions and support to those with mild to moderate mood difficulties poststroke, providing they have suitable training (Intercollegiate Stroke Working Party, 2016; Kneebone, 2016). A promising model may be collaborative working and sharing of skills between SLTs and mental health professionals, so that even those hardest to reach, with both severe aphasia and severe mood difficulties, may be given appropriate psychological support.

Participant perceptions around valued therapy components support the DPM (Stroebe & Schut, 1999) as a framework for conceptualizing the “active ingredients” of the therapy within the SOFIA Trial. Participants valued both loss and restoration-oriented work, as well as “Time Out.” Furthermore, there seemed to be benefit in oscillating between components as predicted by the model.

To turn first to the “restoration work,” participants reported finding it useful to have the opportunity to explore their hopes, as well as notice their own achievements and successes. This matches other research, which has found that hope is a “critical resource for people with aphasia,” sustaining them through uncertain times and creating a sense of possibility for constructing a poststroke life (Bright et al., 2020). Instilling hope, through noticing progress, noticing the person, and noticing their hopes for new possibilities, rather than defining them through their linguistic deficits, may be a helpful focus for clinical interactions (Bright et al., 2020; Lawton et al., 2018). Other research exploring SFBT with clients living with chronic health conditions has also described how the approach can instill hope (Carr et al., 2014; Froerer et al., 2009).

A criticism leveled at SFBT is that, through focusing on solutions, there may be a lack of acknowledgment of the difficulties a person is experiencing (Thomas, 2007). Within this study, acknowledgment, or “grief-oriented work,” was defined as a key component of the intervention. Feedback from participants suggests that many valued this interleaving of possibility with acknowledgment and found it helpful to be able to explore more difficult emotions in a safe space. This matches the findings of the proof-of-concept study, where participants also described the value in being listened to holistically and being able to talk about the challenges of living with stroke and aphasia (Northcott et al., 2015). Similarly, in a nurse-led psychosocial intervention, participants with aphasia reported finding benefit in “narrating about themselves and their experiences with illness.” (Bronken, Kirkevold, Martinsen, Wyller, et al., 2012).

The final element of the DPM model is “Time Out,” endorsed by many participants as a valued therapy component. The DPM model suggests that time out provides a useful reprieve from grief. Having fun perhaps also aligns with what some people with aphasia want from therapy. In a study exploring people with aphasia’s experiences of co-constructing personal narrative within therapy, a theme to emerge was “having fun” (Strong et al., 2018). Similarly, when people with aphasia were invited to codesign a virtual therapy world, their involvement “led to a strong shift from the functional (e.g., a clinic) to the playful (e.g., elephants and mermaids)” (Wilson et al., 2015, p32). Another function of “time out” may be connecting with the therapist. In a study exploring what social support is most valued poststroke, everyday “chit chat” and laughter emerged as a theme in enabling people to feel connected to others (Northcott & Hilari, 2017). It has been argued that professional values instilled in SLTs emphasize professional objectivity and distance, leading to a narrow range of tasks and conversations considered appropriate for therapy interactions (Simmons-Mackie & Damico, 2011). SLTs have been observed to use a variety of strategies to deflect emotional connection with clients to maintain this professional distance (Simmons-Mackie & Damico, 2011). Yet there is increasing evidence that people with aphasia instead value “emotional proximity” with their therapist, including a sense that the therapist is genuine, nonjudgmental, caring, and is “seeing the person” rather than the impairment (Lawton et al., 2018). People with aphasia have been observed to engage more fully in rehabilitation when they perceived that the health care worker prioritized getting to know them, including finding out what mattered to them, and learning about their values, personality, and concerns (Bright et al., 2018).

One of the purposes of conducting qualitative research within a trial is to enable interpretation of the variation in outcomes (Johnson & Schoonenboom, 2016; O’Cathain et al., 2013). To help conceptualize variation, we created a typology, sectoring participants into four discrete groups. The group for whom the therapy was perceived to have the most meaningful impact was the “Changed” group. Many participants in the “changed” group were more than 3 years poststroke, suggesting that people with chronic aphasia may benefit from a linguistically accessible psychological intervention. Given that psychological support in the long-term poststroke has been identified as weak (National Audit Office, 2010), and the high levels of long-term depression poststroke and aphasia (Hackett & Pickles, 2014), this is a concerning gap in current provision.

An unanticipated category was “Companionship.” People with aphasia are at risk of becoming isolated and having reduced social networks (Northcott, Marshall, & Hilari, 2016), which may explain why so many participants valued the companionable aspects of the therapy above all. Furthermore, this matches the stated reason that many people gave for participating in the trial. As the main value for this group was connection rather than creating change, they had less reason to consider therapy as “completed.” This group was also more likely to view the therapist as a friend. It is perhaps unsurprising, therefore, that these participants sometimes found the ending difficult. Sherratt and Hersh (2010) have also observed the challenges around boundaries and endings, particularly when therapy is characterized by partnership and emotional connection, leading them to recommend reflective awareness and ethical problem-solving to protect and support all involved. Consideration could be given to what further training and support SLTs need to enable them to support their clients when therapy ends. For the “Companionship” group, blending the ending of the therapy with establishing new social connections may also be beneficial, for example, through peer befriending (Hilari et al., 2019) or linking to social assets within their local community (Shiggins et al., 2020).

The group that perceived least benefit were the “Discordant” group, who were dissatisfied not to receive “traditional” language therapy. There was potentially a tension between expectations of what therapy would be delivered by an SLT and the therapy delivered within SOFIA. The approach may also have been perceived as more useful for this group had it been integrated with language impairment-based therapy. Where SLTs use SFBT in clinical practice, they report blending it with other SLT therapy approaches (Northcott et al., 2018).

It is noticeable that participant motivation to participate in the trial rarely corresponded with the stated aim of the study (enhancing well-being): They participated to contribute to society, out of curiosity or loneliness, or to improve their language. Furthermore, many participants did not have low mood or well-being. SFBT was developed as an approach to enable people to build change in their lives (Ratner et al., 2012); clinical trials are designed to evaluate change (Johnson & Schoonenboom, 2016) and stroke rehabilitation prioritizes measuring change to evaluate therapy success (Intercollegiate Stroke Working Party, 2016). Yet many participants described how “change” was not what mattered to them about the intervention. What they perceived as important was that the therapeutic interactions made them feel “human,” valued, and noticed as people. Others have argued that, through embracing frameworks such as the humanizing values framework (Galvin & Todres, 2012), stroke care may become more person-centered and holistic (Pound & Greenwood, 2016), enabling health care workers to accord value to interactions that may make a profound difference to their patients, but do not necessarily lead to easy-to-measure change.

### Strengths and Limitations

A main aim of the SOFIA Trial was to explore acceptability of the intervention: This qualitative study has addressed this question and enabled nuanced interpretation of how the intervention was valued. A strength of the study is that people with severe aphasia were enabled to participate in both the therapy and interviews. All 30 participants who received the intervention agreed to be interviewed, providing some reassurance that a variety of perspectives have been elicited. Nonetheless, a potential source of bias is that the lead analyst was also the principal investigator and one of the interviewers. To counter this, the interviewers stressed that they were interested in hearing participants’ honest reflections including negative appraisals, and a second analyst was involved in the analysis. An aphasia screening test, rather than a more comprehensive aphasia assessment, was used to minimize participant burden. SOFIA therapists reported that there were challenges in adapting the therapy for people with more fluent aphasia (reported elsewhere), however, aphasia type was not formally assessed. Another weakness of the study design was that family members were not also participants. As some family members participated in the therapy, they would likely have given further insights into the therapy process and the impact it had on them.

Participants in the intervention group were interviewed 3 months after finishing the intervention. It was anticipated they would be able to reflect on any long-term impact of therapy. However, a disadvantage was that some participants reported difficulty in remembering details about the intervention. Nonetheless, the themes that emerged were similar for both the wait-list group (who were interviewed immediately post intervention), and the intervention group.

### Future Directions

This qualitative study suggests that adapted SFBTis an acceptable approach, potentially warranting further investigation in a definitive trial. Implications for the future trial from this study include enabling potential participants to have a clearer understanding of the therapy approach to manage expectations around language recovery; considering how to target those most likely to gain benefit from the therapy; reflecting on how best to manage endings, including reconsidering the training and support provided to the therapists, and exploring ongoing support options, particularly for those in the “Connected” group.

Mental health professionals are key to provision of mental health services, yet have been described as feeling uncomfortable and inexperienced providing psychological treatments to people with aphasia (Baker et al., 2021). Further research is needed to explore what training and support would best enable mental health professionals to routinely provide aphasia-accessible care across the stroke pathway. Furthermore, brief psychological therapies have successfully been delivered by nurses, occupational therapists, and other members of the stroke multidisciplinary team (Auton et al., 2016; Kitzmüller et al., 2019), including to people with aphasia, supported by an SLT (Bronken, Kirkevold, Martinsen, & Kvigne, 2012). Research could explore collaborative working models, where SLTs support and work with the multidisciplinary team to ensure that psychological care is fully inclusive for people with aphasia.

### Conclusion

It was feasible to adapt SFBT, so that it was acceptable to people with aphasia, including those with a severe communication disability. The approach facilitated many participants to achieve meaningful change, including in their mood and identity. Participants valued being able to explore their hopes, share feelings and achievements, and the companionship and connection they felt with their therapist. There appeared to be value in therapy interactions that enabled participants to feel noticed and validated as people.

## Supplemental Material

sj-pdf-1-qhr-10.1177_10497323211020290 – Supplemental material for “Now I Am Myself”: Exploring How People With Poststroke Aphasia Experienced Solution-Focused Brief Therapy Within the SOFIA Trial Click here for additional data file. Supplemental material, sj-pdf-1-qhr-10.1177_10497323211020290 for “Now I Am Myself”: Exploring How People With Poststroke Aphasia Experienced Solution-Focused Brief Therapy Within the SOFIA Trial by Sarah Northcott, Alan Simpson, Shirley Thomas, Rachel Barnard, Kidge Burns, Shashivadan P. Hirani and Katerina Hilari in Qualitative Health Research

sj-pdf-2-qhr-10.1177_10497323211020290 – Supplemental material for “Now I Am Myself”: Exploring How People With Poststroke Aphasia Experienced Solution-Focused Brief Therapy Within the SOFIA Trial Click here for additional data file. Supplemental material, sj-pdf-2-qhr-10.1177_10497323211020290 for “Now I Am Myself”: Exploring How People With Poststroke Aphasia Experienced Solution-Focused Brief Therapy Within the SOFIA Trial by Sarah Northcott, Alan Simpson, Shirley Thomas, Rachel Barnard, Kidge Burns, Shashivadan P. Hirani and Katerina Hilari in Qualitative Health Research

sj-pdf-3-qhr-10.1177_10497323211020290 – Supplemental material for “Now I Am Myself”: Exploring How People With Poststroke Aphasia Experienced Solution-Focused Brief Therapy Within the SOFIA Trial Click here for additional data file. Supplemental material, sj-pdf-3-qhr-10.1177_10497323211020290 for “Now I Am Myself”: Exploring How People With Poststroke Aphasia Experienced Solution-Focused Brief Therapy Within the SOFIA Trial by Sarah Northcott, Alan Simpson, Shirley Thomas, Rachel Barnard, Kidge Burns, Shashivadan P. Hirani and Katerina Hilari in Qualitative Health Research

sj-pdf-4-qhr-10.1177_10497323211020290 – Supplemental material for “Now I Am Myself”: Exploring How People With Poststroke Aphasia Experienced Solution-Focused Brief Therapy Within the SOFIA Trial Click here for additional data file. Supplemental material, sj-pdf-4-qhr-10.1177_10497323211020290 for “Now I Am Myself”: Exploring How People With Poststroke Aphasia Experienced Solution-Focused Brief Therapy Within the SOFIA Trial by Sarah Northcott, Alan Simpson, Shirley Thomas, Rachel Barnard, Kidge Burns, Shashivadan P. Hirani and Katerina Hilari in Qualitative Health Research

sj-pdf-5-qhr-10.1177_10497323211020290 – Supplemental material for “Now I Am Myself”: Exploring How People With Poststroke Aphasia Experienced Solution-Focused Brief Therapy Within the SOFIA Trial Click here for additional data file. Supplemental material, sj-pdf-5-qhr-10.1177_10497323211020290 for “Now I Am Myself”: Exploring How People With Poststroke Aphasia Experienced Solution-Focused Brief Therapy Within the SOFIA Trial by Sarah Northcott, Alan Simpson, Shirley Thomas, Rachel Barnard, Kidge Burns, Shashivadan P. Hirani and Katerina Hilari in Qualitative Health Research

sj-pdf-6-qhr-10.1177_10497323211020290 – Supplemental material for “Now I Am Myself”: Exploring How People With Poststroke Aphasia Experienced Solution-Focused Brief Therapy Within the SOFIA Trial Click here for additional data file. Supplemental material, sj-pdf-6-qhr-10.1177_10497323211020290 for “Now I Am Myself”: Exploring How People With Poststroke Aphasia Experienced Solution-Focused Brief Therapy Within the SOFIA Trial by Sarah Northcott, Alan Simpson, Shirley Thomas, Rachel Barnard, Kidge Burns, Shashivadan P. Hirani and Katerina Hilari in Qualitative Health Research

sj-pdf-7-qhr-10.1177_10497323211020290 – Supplemental material for “Now I Am Myself”: Exploring How People With Poststroke Aphasia Experienced Solution-Focused Brief Therapy Within the SOFIA Trial Click here for additional data file. Supplemental material, sj-pdf-7-qhr-10.1177_10497323211020290 for “Now I Am Myself”: Exploring How People With Poststroke Aphasia Experienced Solution-Focused Brief Therapy Within the SOFIA Trial by Sarah Northcott, Alan Simpson, Shirley Thomas, Rachel Barnard, Kidge Burns, Shashivadan P. Hirani and Katerina Hilari in Qualitative Health Research

sj-pdf-8-qhr-10.1177_10497323211020290 – Supplemental material for “Now I Am Myself”: Exploring How People With Poststroke Aphasia Experienced Solution-Focused Brief Therapy Within the SOFIA Trial Click here for additional data file. Supplemental material, sj-pdf-8-qhr-10.1177_10497323211020290 for “Now I Am Myself”: Exploring How People With Poststroke Aphasia Experienced Solution-Focused Brief Therapy Within the SOFIA Trial by Sarah Northcott, Alan Simpson, Shirley Thomas, Rachel Barnard, Kidge Burns, Shashivadan P. Hirani and Katerina Hilari in Qualitative Health Research
