# Supplementary material for: “Now I Am Myself”: Exploring How People With Poststroke Aphasia Experienced Solution-Focused Brief Therapy Within the SOFIA Trial
Source: Qual Health Res. 2021 Jun 15;31(11):2041–55. doi: 10.1177/10497323211020290 (PMC8552370; doi:10.1177/10497323211020290)
Supplement: sj-pdf-1-qhr-10.1177_10497323211020290 – Supplemental material for “Now I Am Myself”: Exploring How People With Poststroke Aphasia Experienced Solution-Focused Brief Therapy Within the SOFIA Trial [file sj-pdf-1-qhr-10.1177_10497323211020290.pdf]

Supplemental File 1. An Illustrative Case Example of Solution Focused Brief Therapy

Background information

Geoffrey had a stroke several years prior to participating in SOFIA. He was retired, living on his own, and perceived himself as isolated. He described living with aphasia as traumatic (*'I know everything but the speech, the, I don't know, I, I, difficult, very difficult, it's impossible, impossible'*).

Key therapy activities

Learning what was important to Geoffrey

In a first session, an SFBT therapist will typically explore what their client is hoping for from the therapy as a way of establishing a meaningful focus for sessions. Aware of Geoffrey's anxiety, the therapist chose to spend the first session inviting Geoffrey to notice his competence as a communicator and learning what was important to him. The therapist used picture resources to explore Geoffrey's likes / dislikes, which led to a conversation around the beauty of Bach's violin music. Geoffrey also spent the first session describing his traumatic early hospital experiences post stroke. When asked in the second session about his 'best hopes' from therapy, this led to further discussion of his frustration and anxiety, including descriptions of distressing and unsuccessful conversations. It was only in the third session that the therapist judged that Geoffrey felt sufficiently 'heard' to begin to think about how he wanted to move forwards. She asked him what he wanted instead of feeling anxious. He replied 'calm', which framed many of the conversations in the remaining sessions.

Inviting Geoffrey to notice his own strategies; visualising his preferred future

SFBT has a belief that all clients have resources, and that enabling them to notice their own successful strategies will be more likely to lead to sustainable change. The therapist invited Geoffrey to notice the times when he felt calmer. Geoffrey's list included listening to music, being methodical (for example, in how he went shopping), walking in green spaces, calming the speed of his thinking, slowing his breathing.

Another SFBT tool is inviting a client to describe their preferred future. Geoffrey described how tomorrow would look if he were calm. This included what a 'calm' breakfast would look like, going for a walk and feeling the breeze on his face, how he would know he was calm when he went to his stroke group.

Adapting SFBT to be aphasia-accessible

The therapist wrote down key words, simplified her own language, used pictures, and encouraged Geoffrey to communicate using all modalities, such as gesture and drawing. She

accepted that what might typically occur in a single SFBT session would likely take several sessions. She gave Geoffrey the space and time he needed.

Geoffrey's perspective: illustration of main themes

Geoffrey was interviewed three months after his final therapy session.

Valued therapy components

Geoffrey reported that therapy included describing his successes, such as feeling confident shopping; exploring his hopes; and sharing his anxiety and frustration. He described feeling comfortable to talk with the therapist and perceived these conversations as important to him.

Perceived impact

Geoffrey demonstrated with hand movements that his mood was low pre-therapy (below the desk) and climbed through the therapy. At three months post therapy he indicated that it had stabilised. While he still felt anxious at times, he explained *'current, easier'* than pre-therapy.

Typology category

Changed

In order to preserve anonymity, identifying details have been removed or altered, and a pseudonym used.
