# Supplementary material for: “Now I Am Myself”: Exploring How People With Poststroke Aphasia Experienced Solution-Focused Brief Therapy Within the SOFIA Trial
Source: Qual Health Res. 2021 Jun 15;31(11):2041–55. doi: 10.1177/10497323211020290 (PMC8552370; doi:10.1177/10497323211020290)
Supplement: sj-pdf-2-qhr-10.1177_10497323211020290 – Supplemental material for “Now I Am Myself”: Exploring How People With Poststroke Aphasia Experienced Solution-Focused Brief Therapy Within the SOFIA Trial [file sj-pdf-2-qhr-10.1177_10497323211020290.pdf]

Supplemental File 2. Questions to assess capacity

1. Will the researchers visit you **once** or **several times**?

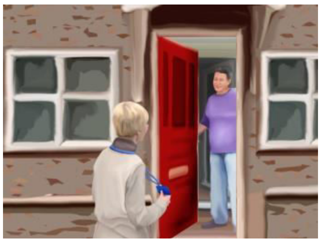

Once	Several times

2. Can you **stop** if you wish, **yes** or **no**?

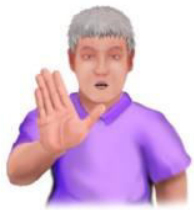

Yes	No

3. Is this study about a **new drug** OR involve **talking** to a Speech and Language Therapist?

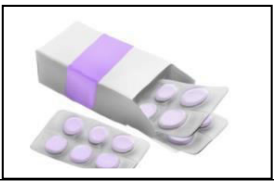	New drug

OR

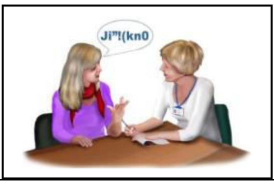	Talking
