# Supplementary material for: “Now I Am Myself”: Exploring How People With Poststroke Aphasia Experienced Solution-Focused Brief Therapy Within the SOFIA Trial
Source: Qual Health Res. 2021 Jun 15;31(11):2041–55. doi: 10.1177/10497323211020290 (PMC8552370; doi:10.1177/10497323211020290)
Supplement: sj-pdf-3-qhr-10.1177_10497323211020290 – Supplemental material for “Now I Am Myself”: Exploring How People With Poststroke Aphasia Experienced Solution-Focused Brief Therapy Within the SOFIA Trial [file sj-pdf-3-qhr-10.1177_10497323211020290.pdf]

Supplemental File 3: Topic guide

Objectives:

1. Explore their experiences of the study, including recruitment and consent procedure, project information, randomisation process, assessment protocol, therapy protocol, ending of involvement in project

2. Suggestions for change to study protocol to inform design of future larger-scale study

3. Explore impact on their life of taking part in the research project (if any) and perceived mechanisms of change (if any change identified)

4. Explore their experiences of the intervention; explore experiences of usual care in the context of receiving SFBT

Pre-interview: reaffirm consent; tape recording

Thank yous: for their time and taking part in the project

Reassurances: confidential; can stop/take a break; no right or wrong answers, interested in their views

Time: about 1 hour

Aim of interview: explore how they found taking part in the study

1. **Complete Session Rating Scale**
2. **Experiences of taking part in the project – recruitment/ assessment *PHOTOS OF RA &THERAPIST***

   Possible opener: want to understand their experiences, so can improve the project. Helpful to hear: what's working, what we should change.

   'Can you tell me about your experience of taking part in this study?'

   - Recruitment procedure/ initial contact/ ongoing communication
     - Initial contact/ how heard about the project
     - Factors that made them decide to take part/ any reservations
     - Initial information about project
     - Group allocation (acceptability, how this was communicated)
     - Ongoing contact – e.g. telephone / email contact with SN

       *** suggestions for change***

- Assessments/questionnaires (their experiences of answering questions/ assessment sessions)
  - General – how did they find the assessment sessions/ overall experience (what was positive/ less positive etc)/
  - Logistics – how organised/ arranged/ location
  - Questionnaires - easy to understand?
  - Length of assessment sessions/fatigue/ too many questionnaires?
  - The right questions? (were the questions relevant to you? Study was about well-being – did the questions capture this?)
  - relationship with RA – and if good, what RA did that made the sessions a positive thing

- How felt about being asked to keep RA 'blinded'
  *** suggestions for change***

- Wait list experience (for wait list group)
  - Acceptability of waiting for therapy input; how this process was communicated etc
  - Whether knowing everyone receives the therapy influenced their decision to participate

3. Experiences of Intervention *(intervention group only)* - *DIAGRAM*

2.1 Overall impression:
How did you find the therapy?

- What worked well (if any)/ perceived as useful (if any)/ what did they like or enjoy (if any)
- What did they find unhelpful? How could we improve therapy?

2.2 Therapy components:

Explore what happened in the therapy sessions (what sorts of things they talked about)

Probe how they found the following components of the therapy - **use diagram as appropriate to scaffold**

Possible therapy components – see diagram:

- *Relationship with therapist*
- *'Your story' (stroke/ recovery)*
- *Moving forwards (hopes for future)*
- *Building on past successes/ 'noticing'*
- *Sharing distress (and someone caring/ listening)*
- *Time out*

'Successful' conversations

Other therapeutic components (e.g. help with practical things)

conversation

2.3 Logistics (arrangements, length of sessions, location)

2.4 Endings the therapy

- Number of sessions
- How ending of therapy was handled
- How they experienced the ending/ weeks after therapy had ended – and what might have helped

2.5 **Best time** to receive SFBT in stroke 'journey'

(stroke: big change in life. Hospital, rehabilitation, then getting used to living with aphasia.)

Best time to have SFBT? (hospital; first home from hospital/ community rehab; at end of all the rehab?; different for everyone?)

2.6 Their experience of combining it with other 'usual care'

-acceptability
-probe how found it having SFBT and usual care where applicable (e.g. SLT)

2.7. Overall evaluation: would they recommend this therapy to someone who's had a stroke?

4. Perceived impact of taking part in the project on their life CALENDAR; PICTURES

Explore what, if anything, has changed over previous 6 months.

General: 'Have you experienced any changes since taking part in the project?' (explore using calendar when first met CI)

Possible areas to probe:

3.1. Person-specific (i.e. related to what was worked on in the therapy – intervention group only)

3.2. Confidence

3.3. Feelings/ mood/ optimism for the future

3.4. Relationships with family and friends

3.4. Communication

3.5. Participation (taking part/ 'doing' things incl work roles)/ everyday life

3.6 Activities of daily living/ independence/ mobility

- Take them back to when started the project. Consider if they feel anything has changed in each area.
- If change has been observed/ perceived, what they think accounts for the change / how do they make sense of it
- Impact of change – if any (e.g. on day to day life/mood)

5. Suggestions/ overall comments

Signpost coming towards end of interview:

- Overall evaluation of the project/ final comments
- Therapy group: How should we describe this therapy to future participants?
  Wait list group: How they would describe this project to others who might want to take part?

Provision of any relevant information<br><br>Check if want to be invited to dissemination event/ be told about results<br><br>Check if want to be told about other City University research projects/ research events/ receive Aphasia Team newsletter<br><br>Reassurances about confidentiality/ what will happen next<br><br>Thank yous!
