# Supplementary material for: “Now I Am Myself”: Exploring How People With Poststroke Aphasia Experienced Solution-Focused Brief Therapy Within the SOFIA Trial
Source: Qual Health Res. 2021 Jun 15;31(11):2041–55. doi: 10.1177/10497323211020290 (PMC8552370; doi:10.1177/10497323211020290)
Supplement: sj-pdf-4-qhr-10.1177_10497323211020290 – Supplemental material for “Now I Am Myself”: Exploring How People With Poststroke Aphasia Experienced Solution-Focused Brief Therapy Within the SOFIA Trial [file sj-pdf-4-qhr-10.1177_10497323211020290.pdf]

Supplemental File 4. Participant characteristics

Characteristic		Participant numbers (%)
**Gender**	Female	16 (53.3%)
	Male	14 (46.7%)
**Age**	Mean (S.D.): 67.7 (12.67); Range: 35-86	
**Ethnicity**	Asian	5 (16.7%)
	Black	3 (10%)
	White	22 (73.3%)
**Living situation**	Living with family	18 (60%)
	Living alone	10 (33.3%)
	Living with carer/ living in sheltered housing	2 (6.7%)
**Time post onset**	<12 mths	8 (26.7%)
	1-2 yrs	9 (30%)
	>2 yrs	13 (43.3%)
**Aphasia severity***	Mild-Moderate	16 (53.3%)
	Severe	14 (46.7%)
**Frenchay Aphasia Screening Test scores (range: 0-30, higher scores indicate milder aphasia)**		
**Mean (s.d.)**	15.47 (8.35)	n=30
**Median (IQR)**	16.50 (7.75, 22.25)	n=30

*Participants scoring <7/15 on either the receptive or expressive domains of the Frenchay Aphasia Screening Test (Enderby et al., 1987) categorised as 'severe' within SOFIA Trial.
