# Supplementary material for: “Now I Am Myself”: Exploring How People With Poststroke Aphasia Experienced Solution-Focused Brief Therapy Within the SOFIA Trial
Source: Qual Health Res. 2021 Jun 15;31(11):2041–55. doi: 10.1177/10497323211020290 (PMC8552370; doi:10.1177/10497323211020290)
Supplement: sj-pdf-5-qhr-10.1177_10497323211020290 – Supplemental material for “Now I Am Myself”: Exploring How People With Poststroke Aphasia Experienced Solution-Focused Brief Therapy Within the SOFIA Trial [file sj-pdf-5-qhr-10.1177_10497323211020290.pdf]

Supplemental File 5: individual participant profiles, organised by typology category

Participant	Baseline WEMWBS	Aphasia severity	FAST total	TPO at Baseline	Therapist	Number of sessions	Trial Arm
**'Changed'**: meaningful impact (n=11)							
#1	Low-average	Mild-moderate	28	>3 years	SLT2	6	Intervention
#2	Low-average	Mild-moderate	25	1-2 years	SLT3	6	Wait-list
#3	High	Mild-moderate	18	>3 years	SLT2	6	Intervention
#4	Low-average	Mild-moderate	18	<12 months	SLT1	6	Wait-list
#5	Low-average	Mild-moderate	15	>3 years	SLT1	6	Intervention
#6	High	Mild-moderate	22	1-2 years	SLT1	6	Wait-list
#7	Low-average*	Severe	9	<12 months	SLT3	6	Wait-list
#8	Low-average	Mild-moderate	22	>3 years	SLT1	6	Wait-list
#9	Low-average	Severe	3	>3 years	SLT1	6	Wait-list
#10	Low-average	Mild-moderate	14	1-2 years	SLT2	6	Intervention
#11	Low-average	Severe	2	>3 years	SLT1	6	Intervention
**'Connected'**: connection and companionship (n=10)							
#12	High	Mild-moderate	25	>3 years	SLT3	6	Intervention
#13	High	Severe	13	<12 months	SLT2	6	Wait-list
#14	High	Severe	5	1-2 years	SLT1	6	Intervention
#15	High	Mild-moderate	18	>3 years	SLT2	6	Wait-list
#16	High	Mild-moderate	22	1-2 years	SLT3	6	Intervention
#17	Low-average	Severe	9	<12 months	SLT3	6	Wait-list
#18	Low-average*	Mild-moderate	28	1-2 years	SLT3	6	Wait-list
#19	High	Severe	13	>3 years	SLT1	6	Intervention
#20	Low-average	Severe	7	<12 months	SLT1	6	Intervention
#21	High	Severe	7	>3 years	SLT2	6	Intervention
**'Complemental'**: complementing an upward trajectory (n=4)							
#22	High	Severe	4	<12 months	SLT1	5	Intervention
#23	Low-average	Severe	9	<12 months	SLT1	6	Intervention
#24	High	Severe	4	1-2 years	SLT3	6	Wait-list
#25	Low-average	Severe**	20	<12 months	SLT2	6	Wait-list
**'Discordant':** dissatisfied with focus of therapy (n=5)							
#26	Low-average	Mild-moderate	25	1-2 years	SLT3	6	Wait-list
#27	Low-average	Mild-moderate	27	>3 years	SLT1	6	Intervention
#28	Low-average	Severe	8	<12 months	SLT3	6	Intervention
#29	Low-average	Mild-moderate	23	>3 years	SLT3	6	Intervention
#30	Low-average	Mild-moderate	21	2-3 years	SLT3	6	Wait-list

FAST: Frenchay Aphasia Screening Test, range: 0-30, higher scores indicate milder aphasia. Participants categorised as 'severe' if scored <7/15 on either receptive or expressive domains.
WEMWBS = Warwick Edinburgh Mental Wellbeing Scale, classified as 'high' if score ≥59/70;
TPO = Time Post Onset; SLT = Speech and Language Therapist;
*changed category between baseline assessment and start of therapy, applies only to wait-list group.
**participant had severe expressive aphasia but excellent receptive skills.
