# Supplementary figures and images for: “Now I Am Myself”: Exploring How People With Poststroke Aphasia Experienced Solution-Focused Brief Therapy Within the SOFIA Trial
Source: Qual Health Res. 2021 Jun 15;31(11):2041–55. doi: 10.1177/10497323211020290 (PMC8552370; doi:10.1177/10497323211020290)

## Supplemental File 6: SOFIA CONSORT Diagram

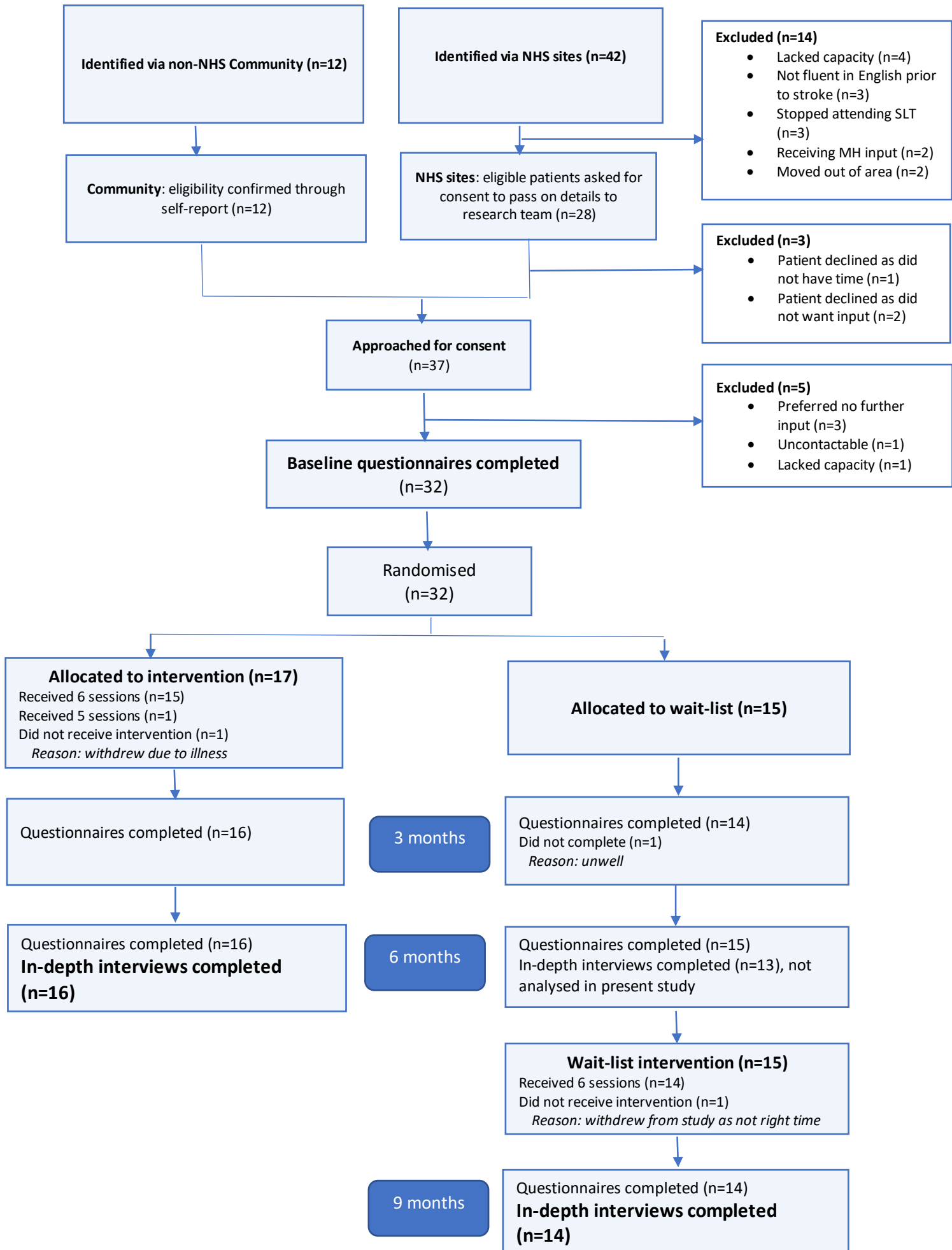

Supplement: sj-pdf-6-qhr-10.1177_10497323211020290 – Supplemental material for “Now I Am Myself”: Exploring How People With Poststroke Aphasia Experienced Solution-Focused Brief Therapy Within the SOFIA Trial [file sj-pdf-6-qhr-10.1177_10497323211020290.pdf]

## Supplemental File 7. Prevalence of the therapy components within participants' accounts

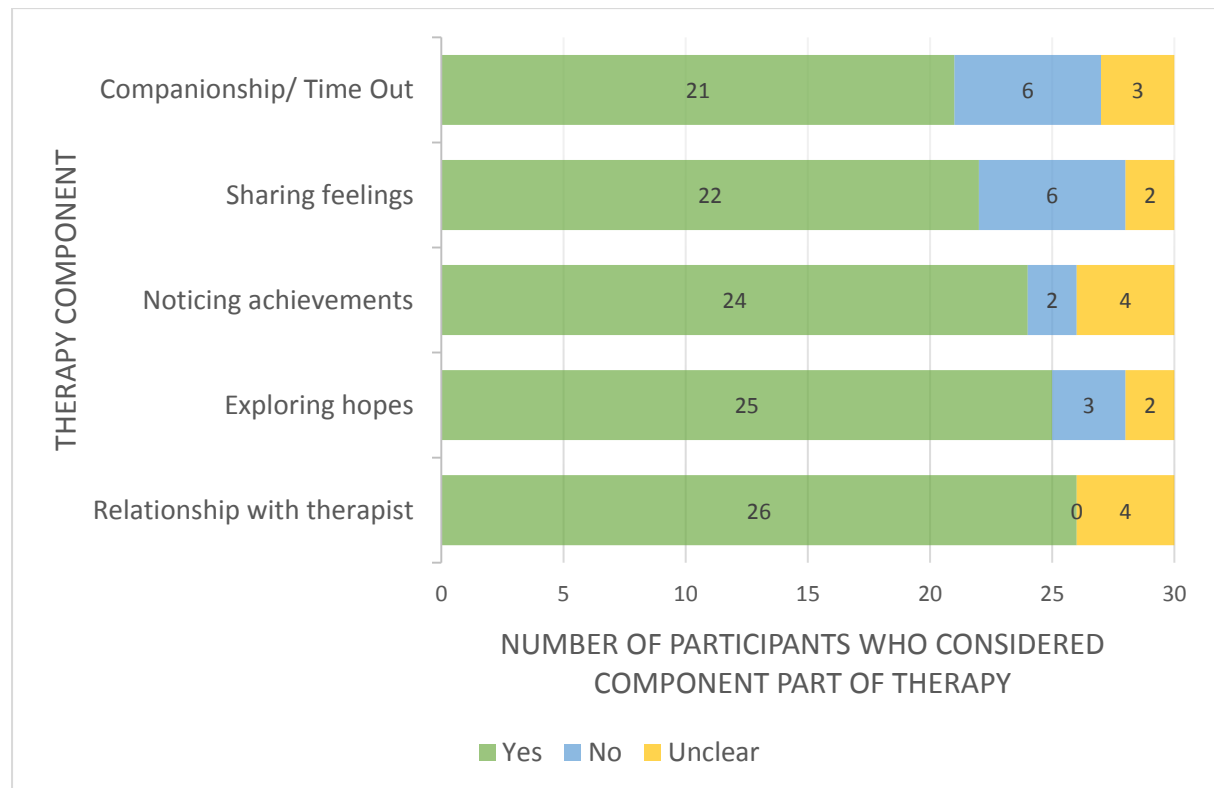

Supplement: sj-pdf-7-qhr-10.1177_10497323211020290 – Supplemental material for “Now I Am Myself”: Exploring How People With Poststroke Aphasia Experienced Solution-Focused Brief Therapy Within the SOFIA Trial [file sj-pdf-7-qhr-10.1177_10497323211020290.pdf]
