# Supplementary material for: “Now I Am Myself”: Exploring How People With Poststroke Aphasia Experienced Solution-Focused Brief Therapy Within the SOFIA Trial
Source: Qual Health Res. 2021 Jun 15;31(11):2041–55. doi: 10.1177/10497323211020290 (PMC8552370; doi:10.1177/10497323211020290)
Supplement: sj-pdf-8-qhr-10.1177_10497323211020290 – Supplemental material for “Now I Am Myself”: Exploring How People With Poststroke Aphasia Experienced Solution-Focused Brief Therapy Within the SOFIA Trial [file sj-pdf-8-qhr-10.1177_10497323211020290.pdf]

Supplemental File 8: Core themes, subthemes and typology

Core theme	Sub-theme
1. Valued therapy components	a) Being facilitated to notice personal qualities and achievements<br>b) Encouragement to explore hopes for the future<br>c) Feeling supported to explain how they feel<br>d) Companionship and 'time out'<br>e) Relationship with therapist
2. Perceptions around progress	a) Mood and identity<br>b) Communication<br>c) Relationships<br>d) Independence, mobility and participation<br>e) Not changing
3. Experiencing therapy within a research project	a) Motivations for participating in study<br>b) Constraints on therapy offered

Typology (categorisation based on perceived value of therapy and therapy-related change)

Category	*Relationship to themes*
**Changed** (n=11; 27% severe aphasia)	Therapy highly valued (Theme 1); perceived to lead to meaningful change (Theme 2a-d)
**Connected** (n=10; 60% severe aphasia)	Therapy valued primarily for companionship (Theme 1d&e, often facilitated by Theme 1a&c); participants not seeking to make change (Theme 2e)
**Complemental** (n=4; 100% severe aphasia)	Therapy perceived as positive experience (Theme 1), and complemented participants' upward trajectory (Theme 2).
**Discordant** (n=5; 20% severe aphasia)	Therapy not aligned with participant's preferred focus on language impairment (Theme 3a). Although most described some progress (e.g. Theme 2a&d), they were dissatisfied with language recovery (Theme 2e).
